# The impact of sex and age on neurological outcomes in out-of-hospital cardiac arrest patients with targeted temperature management

**DOI:** 10.1186/s13054-017-1860-5

**Published:** 2017-11-02

**Authors:** Sang Hoon Oh, Kyu Nam Park, Jeeyong Lim, Seung Pill Choi, Joo Suk Oh, In Soo Cho, Byung Kook Lee, Yong Hwan Kim, Young-Min Kim, Han Joon Kim, Chun Song Youn, Soo Hyun Kim, On behalf of the Korean Hypothermia Network Investigators

**Affiliations:** 1 Department of Emergency Medicine, College of Medicine, Seoul St. Mary’s Hospital, The Catholic University of Korea, 222 Banpo-daero, Seocho-gu, Seoul 06591 Republic of Korea; 2 Department of Emergency Medicine, KEPCO Medical Center, Seoul, South Korea; 3 Department of Emergency Medicine, College of Medicine, Chonnam National University, Gwangju, South Korea; 4 Department of Emergency Medicine, Samsung Changwon Hospital, Sungkyunkwan University School of Medicine, Changwon, South Korea

**Keywords:** Out-of-hospital cardiac arrest, Sex, Age groups, Induced hypothermia

## Abstract

**Background:**

There are conflicting data regarding sex-based differences in the outcomes of out-of-hospital cardiac arrest (OHCA) patients, and whether the specific sex advantage is age-specific remains unclear. We assessed the impact of the interactions between sex and age on the neurological outcomes of OHCA patients receiving targeted temperature management (TTM).

**Methods:**

Data collected from 2007 to 2012 for a multicenter, registry-based study of the Korean Hypothermia Network were analyzed. We used a multivariate logistic regression model with an interaction term (age × sex) as the final model for the outcomes. To evaluate the association between sex and outcome in specific age groups, all patients were divided into specific age subgroups, and the adjusted ORs and 95% CIs of good neurological outcomes for males were calculated for each age group. Finally, the ORs of a good neurological outcome for the specific age groups compared with the 50- to 59-year-old group were calculated for both sexes.

**Results:**

In the interaction analysis, age was a negative prognostic factor (OR, 0.95 [95% CI, 0.93-0.98]), whereas sex was not associated with neurological outcomes (OR, 3.74 [95% CI, 0.85–16.35]), and reproductive age in females (age, < 50 years) was also not associated with good neurological outcomes. After the patients were divided into five age groups, sex was not an independent predictor of neurological outcomes across all age groups. Patients of both sexes aged < 40 years had significantly better outcomes than patients in the 50- to 59-year-old group (males, OR, 4.03 [95% CI, 1.86–8.73]; females, OR, 10.34 [95% CI, 1.99–53.85]). Males aged ≥ 70 years had significantly poorer neurological outcomes than those in the 50- to 59-year-old group (OR, 0.15 [95% CI, 0.07–0.32]), but this outcome was not observed for females (OR, 0.78 [95% CI, 0.20–3.14]).

**Conclusions:**

Sex did not influence the neurological outcomes of TTM-treated OHCA patients. In contrast to the outcomes in males, the neurological outcomes of females worsened from 18 to 59 years of age and then remained constant.

## Background

In out-of-hospital cardiac arrest (OHCA) patients, there is evidence that survival varies with patient sex and age [1–11]. Generally, old age is considered a negative prognostic factor after OHCA [1]. Reports regarding sex-based differences in outcomes are conflicting, with some studies showing comparable survival in females and males [4, 5] and other studies showing comparable but better survival in females of reproductive age [6–10]. A recent meta-analysis of observational studies demonstrated an association between female sex and increased likelihood of survival [11].

Several interventions have been shown to improve outcomes following cardiac arrest. In particular, targeted temperature management (TTM) is now recommended as standard care in these patients [12]. However, the majority of previous studies regarding sex and age differences in outcomes included the entire emergency medical service (EMS)-attended cardiac arrest population and did not examine TTM-treated patients. Importantly, these data did not include postresuscitation neurological outcomes. Although some studies enrolled these patients, they were not focused on interactions between patient sex and age [13–16]. Therefore, whether sex affects neurological outcomes after TTM and whether the specific sex advantage is age-specific remain unclear.

In the present study, we analyzed Korean Hypothermia Network (KORHN) registry data obtained from 930 OHCA cases with TTM. The purpose of the present study was to describe the impact of sex and age on the neurological outcomes of OHCA patients with TTM. We assessed the effect of sex and age using logistic regression with interaction analysis. We also divided the patients into age subgroups and examined age-specific differences in the outcomes between sex and age subgroups.

## Methods

### Study design

This study was a multicenter, retrospective, observational, registry-based study that used KORHN registry data. The KORHN investigators collected data on adult (age ≥ 18 years) OHCA patients who received TTM and advanced critical care in 24 teaching hospitals in South Korea from 2007 to 2012. Patients who experienced traumatic cardiac arrest were excluded. The data form, the standard definitions of 87 variables, and the registration manual were developed after a literature review and via the consensus of the study investigators. The registry data were collected from medical charts or electronic medical record reviews. The protocols of all centers included the same TTM parameters: a target temperature of 33 °C, 24-h maintenance, and controlled normothermia for 72 h after the return of spontaneous circulation (ROSC). The KORHN study design, including primary and other outcomes, was published previously [17, 18]. The KORHN registry study protocol was approved by the ethics committees in each participating hospital, and informed consent was waived.

This study included all patients who received TTM. We analyzed sex, age, and possible covariates for neurological outcomes, such as other demographic and resuscitation variables, which were collected according to the Utstein guidelines [19]. Comorbidities were registered if they were pharmacologically or previously surgically treated or if they were subject to continuous supervision at the time of cardiac arrest.

The primary outcome was good neurological outcome, which was evaluated by attending physicians or independent neurologists and categorized at hospital discharge according to the Glasgow-Pittsburgh Cerebral Performance Categories (CPCs). The outcomes were dichotomized as good (CPC 1, 2) and poor (CPC 3–5) [20]. The secondary outcome was survival to hospital discharge.

### Statistical analysis

Categorical variables are presented as the total number of patients and the proportion of patients, and continuous variables are reported as the mean and SD. To compare the distribution of the characteristics between two groups, we used the chi-square test for categorical variables and Student’s *t* test for continuous variables.

Logistic regression analysis was used to establish the association of sex and age with good neurological outcome and survival to discharge, and ORs and 95% CIs were estimated. We used a multivariate logistic regression model with an interaction term (age × sex) as the final model for the outcomes. To assess whether there was an association between reproductive age in females and outcomes, we divided the patients into two age subgroups (age < 50 years and ≥ 50 years). To evaluate whether there was a sex advantage in a specific age group, the patients were also divided into five age subgroups (<40 years, 40–49 years, 50–59 years, 60–69 years, and ≥ 70 years), and all variables with a significance level of *p* < 0.05 in the multivariate analysis were included in the adjustment. The adjusted ORs of male sex for good neurological outcomes and survival to discharge were calculated for each age group. Finally, the ORs of good neurological outcomes for specific age groups compared with the 50- to 59-year-old group were calculated for both sexes.

All statistical analyses were performed using IBM SPSS version 24 software (IBM, Armonk, NY, USA). All *p* values were two-tailed, and *p* < 0.05 was considered significant.

## Results

### Characteristics of the study population by sex

**Fig. 1:**
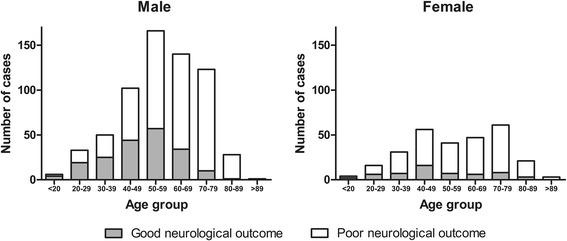
Number of cases treated with targeted temperature management after out-of-hospital cardiac arrest for both sexes

Over the 6-year study period, a total of 930 OHCA patients were entered into the registry, and all these patients were included in the analysis. Of these patients, 650 (70.1%) were male, and the remaining 280 (29.9%) were female. The age-specific enrollment rates are shown in Fig. 1. The male group was most affected in the 50- to 59-year-old age group. In contrast, females showed a bimodal distribution, with the 40- to 49-year-old and 70- to 79-year-old groups most affected.

The baseline characteristics of the study patients according to sex are presented in Table 1. The average ages of the male and female patients were 56.7 ± 15.4 and 56.9 ± 18.1 years, respectively (*p* = 0.854). The percentages of patients with chronic renal disease and liver cirrhosis significantly differed between the male and female groups (4.8% vs. 9.6%, *p* = 0.005; and 1.8% vs. 0.0%, *p* = 0.022, respectively). No significant differences were found in the other underlying comorbidities. In the resuscitation variables, the male patients were significantly more likely to be witnessed during cardiac arrest, to exhibit more shockable rhythms, and to display an increased presumed cardiac cause rate than the female patients (68.9% vs. 62.1%, *p* = 0.044; 33.1% vs. 20.0%, *p* < 0.001; and 64.3% vs. 52.1%, *p* < 0.001, respectively). The anoxic time (time interval from arrest to ROSC) did not significantly differ between the two groups (*p* = 0.296). Early coronary angiography (CAG) within 24 h after ROSC was performed more frequently in males than in females (15.5% vs. 7.9%, *p* = 0.002). The survival discharge and good neurological outcome proportions were significantly higher in the male group than in the female group (63.8% vs. 50.4%, *p* < 0.001; and 29.8% vs. 19.6%, *p* < 0.001, respectively).

**Table 1:** Characteristics of study participants, by sex

	Males (*n* = 650)	Females (*n* = 280)	*p* Value
Age, years	56.7 ± 15.4	56.9 ± 18.1	0.854
Comorbidity			
Coronary artery disease	80 (12.3)	32 (11.4)	0.706
Congestive heart failure	17 (2.6)	12 (4.3)	0.179
Stroke	32 (4.9)	14 (5.0)	0.960
Hypertension	220 (33.8)	101 (36.1)	0.513
Diabetes mellitus	137 (21.1)	72 (25.7)	0.120
Chronic lung disease	37 (5.7)	19 (6.8)	0.520
Chronic renal disease	31 (4.8)	27 (9.6)	0.005
Liver cirrhosis	12 (1.8)	0 (0.0)	0.022
Malignancy	22 (3.4)	5 (1.8)	0.183
Witnessed	448 (68.9)	174 (62.1)	0.044
Bystander CPR	204 (31.4)	77 (27.5)	0.237
Shockable rhythm	215 (33.1)	56 (20.0)	< 0.001
Cardiac cause	418 (64.3)	146 (52.1)	< 0.001
Anoxic time, minutes	34.4 ± 18.5	33.0 ± 16.7	0.296
Early coronary angiography	101 (15.5)	22 (7.9)	0.002
Survival discharge	415 (63.8)	141 (50.4)	< 0.001
Good neurological outcome	194 (29.8)	55 (19.6)	< 0.001

*CPR* Cardiopulmonary resuscitation

Data show the number of cases (percentages)

### Effect of sex and age on neurological outcomes and survival to discharge

To evaluate whether specific sex and age were independent predictors for neurological outcomes, a multivariate logistic regression model was created (Table 2). The model was adjusted for sex, age, bystander-witnessed status, bystander cardiopulmonary resuscitation (CPR) performance, first recorded rhythm during cardiac arrest, presumed arrest cause, anoxic time, and early CAG. In the unadjusted analysis, age, sex, and all other variables were significantly associated with neurological outcomes at hospital discharge.

**Table 2:** Effect of variables, including age and sex, in a logistic regression model and an interaction model

	Univariate analysis			Multivariate analysis^a^			Interaction analysis^b^		
	OR	95% CI	*p* Value	OR	95% CI	*p* Value	OR	95% CI	*p* Value
Good neurological outcome									
Male sex	1.74	1.24–2.44	0.001	1.44	0.93–2.23	0.099	3.74	0.85–16.35	0.080
Age, per year	0.96	0.95–0.97	< 0.001	0.94	0.93–0.96	< 0.001	0.95	0.93–0.98	< 0.001
Witnessed	2.76	1.93–3.94	< 0.001	1.75	1.11–2.77	0.016	1.74	1.10–2.75	0.017
Bystander CPR	1.85	1.37–2.52	< 0.001	0.81	0.53–1.24	0.333	0.81	0.53–1.23	0.316
Shockable rhythm	7.40	5.36–10.22	< 0.001	4.47	2.94–6.79	< 0.001	4.48	2.95–6.82	< 0.001
Cardiac cause	7.78	5.11–11.85	< 0.001	6.00	3.53–10.20	< 0.001	5.95	3.50–10.12	< 0.001
Anoxic time, per minute	0.96	0.95–0.97	< 0.001	0.94	0.93–0.96	< 0.001	0.94	0.93–0.96	< 0.001
Early coronary angiography	2.77	1.87–4.09	< 0.001	1.34	0.81–2.23	0.260	1.31	0.78–2.18	0.303
Age × sex							0.98	0.96–1.01	0.183
Survival to discharge									
Male	1.74	1.31–2.31	0.001	1.71	1.24–2.35	0.001	2.16	0.69–6.76	0.187
Age, per year	0.98	0.97–0.99	< 0.001	0.97	0.96–0.98	< 0.001	0.98	0.96–0.99	0.001
Witnessed	2.23	1.69–2.95	< 0.001	1.86	1.34–2.57	< 0.001	1.86	1.34–2.57	< 0.001
Bystander CPR	1.21	0.91–1.62	0.190	0.68	0.48–0.95	0.026	0.68	0.48–0.95	0.025
Shockable rhythm	3.58	2.57–4.99	< 0.001	2.19	1.48–3.26	< 0.001	2.19	1.47–3.25	< 0.001
Cardiac cause	2.86	2.18–3.76	< 0.001	2.06	1.48–2.87	< 0.001	2.05	1.47–2.86	< 0.001
Anoxic time, per minute	0.98	0.97–0.99	< 0.001	0.98	0.97–0.98	< 0.001	0.98	0.97–0.98	< 0.001
Early coronary angiography	2.00	1.31–3.05	0.001	1.10	0.68–1.80	0.695	1.10	0.67–1.80	0.707
Age × sex							1.00	0.98–1.02	0.673

*CPR* Cardiopulmonary resuscitation

^a^Adjusted for sex, age, bystander-witnessed status, bystander cardiopulmonary resuscitation performance, first recorded rhythm during cardiac arrest, presumed arrest cause, anoxic time, and early coronary angiography

^b^Adjusted for covariates in the multivariate analysis and interaction term (age × sex)

After adjusting for the selected baseline characteristics, witnessed cardiac arrest (OR, 1.75 [95% CI, 1.11–2.77], *p* = 0.016), initial shockable rhythm (OR, 4.47 [95% CI, 2.94–6.79], *p* < 0.001), and presumed cardiac cause (OR, 6.00 [95% CI, 3.53–10.20], *p* < 0.001) were significantly associated with a good neurological outcome. Age and anoxic time were negative prognostic factors (OR, 0.94 [95% CI, 0.93–0.96], *p* < 0.001; and OR, 0.94 [95% CI, 0.93–0.96], *p* < 0.001, respectively). In contrast, male sex (OR, 1.44 [95% CI, 0.93–2.23], *p* = 0.099), bystander CPR, and early CAG were not associated with the neurological outcome. After adjusting for covariates in the interaction model, the ORs of male sex and age for good neurological outcome were 3.74 (95% CI, 0.85–16.35) and 0.95 (95% CI, 0.93–0.98), respectively.

Regarding secondary outcomes, age was associated with in-hospital mortality (OR, 0.97 [95% CI, 0.96–0.98], *p* < 0.001). Male sex was associated with survival to discharge (OR, 1.71 [95% CI, 1.24–2.35], *p* = 0.001), whereas the interaction analysis indicated that male sex was not associated with survival to discharge (OR, 2.16 [95% CI, 0.69–6.76], *p* = 0.187). For both neurological outcomes and survival to discharge, the sex × age interactions were not statistically significant (*p* = 0.183 and *p* = 0.673, respectively).

### Neurological outcome by age group and sex

Table 3 shows the age-stratified outcomes after OHCA with TTM by sex. When we divided the patients into two age groups (age < 50 years, *n* = 298; ≥ 50 years, *n* = 632), the sex effect for good neurological outcomes was statistically nonsignificant in both age groups (*p* = 0.226 and *p* = 0.389, respectively). For survival to discharge, whereas there was no significant difference between sexes in the < 50 years age group, for the age ≥ 50 years group, male sex was significantly associated with survival to discharge (OR, 1.78 [95% CI, 1.21–2.63], *p* = 0.004).

**Table 3:** Outcomes of out-of-hospital cardiac arrest patients after targeted temperature management, by age group and sex

	Good neurological outcome					Survival to discharge				
	Outcome, *n* (%)	Crude OR (95% CI)	*p* Value	Adjusted OR^a^ (95% CI)	*p* Value	Outcome, *n* (%)	Crude OR (95% CI)	*p* Value	Adjusted OR^a^ (95% CI)	*p* Value
Two categorizations										
Age < 50 years										
Male	92 (48.2)	2.28 (1.38–3.78)	0.001	1.52 (0.77–2.98)	0.226	140 (73.3)	1.77 (1.07–2.93)	0.026	1.14 (0.62–2.11)	0.667
Female	31 (29.0)					65 (60.7)				
Age ≥ 50 years										
Male	102 (22.2)	1.77 (1.09–2.88)	0.020	1.29 (0.72–2.33)	0.389	275 (59.9)	1.91 (1.34–2.72)	<0.001	1.78 (1.21–2.63)	0.004
Female	24 (13.9)					76 (43.9)				
Five categorizations										
Age < 40 years										
Male	48 (53.9)	2.81 (1.35–5.85)	0.006	1.34 (0.47–3.84)	0.584	62 (69.7)	1.36 (0.66–2.82)	0.402	0.79 (0.31–2.00)	0.620
Female	15 (29.4)					32 (62.7)				
Age 40–49 years										
Male	44 (43.1)	1.90 (0.94–3.82)	0.073	1.63 (0.65–4.09)	0.297	78 (76.5)	2.27 (1.12–4.57)	0.022	1.48 (0.63–3.43)	0.368
Female	16 (28.6)					33 (58.9)				
Age 50–59 years										
Male	57 (34.3)	2.54 (1.06–6.09)	0.037	2.31 (0.81–6.63)	0.119	118 (71.1)	2.58 (1.28–5.19)	0.008	2.62 (1.21–5.69)	0.015
Female	7 (10.9)					20 (48.8)				
Age 60–69 years										
Male	34 (24.3)	2.19 (0.86–5.61)	0.102	1.25 (0.41–3.81)	0.693	87 (62.1)	2.03 (1.04–3.97)	0.038	2.02 (0.95–4.28)	0.068
Female	6 (12.8)					21 (44.7)				
Age ≥ 70 years										
Male	11 (7.2)	0.52 (0.22–1.26)	0.147	0.56 (0.19–1.63)	0.289	70 (45.8)	1.21 (0.71–2.06)	0.496	1.40 (0.79–2.51)	0.251
Female	11 (12.9)					35 (41.2)				

^a^Adjusted ORs are adjusted for age, bystander-witnessed status, first recorded rhythm during cardiac arrest, presumed arrest cause, and anoxic time

To evaluate whether there was a sex advantage in any specific age group after TTM, the patients were stratified by sex and divided into five age groups as follows: < 40 years, *n* = 140; 40–49 years, *n* = 158; 50–59 years, *n* = 207; 60–69 years, *n* = 187; and ≥ 70 years, *n* = 238. Although in some subgroups males had significantly higher odds of good neurological outcomes than females in the univariate analysis, the multivariate analysis revealed no significant differences in neurological outcomes between sexes across all age groups. The trend of change in the adjusted ORs for male sex increased with age from < 40 years to 50–59 years. Males aged 50–59 years had a peak OR that decreased as they aged. Finally, males aged > 70 years had a lower rate of good neurological outcomes than females (7.2% vs. 12.9%) and were more likely to be associated with a poor neurological outcome (OR, 0.56 [95% CI, 0.19-1.63], *p* = 0.289), although the difference was not significant.

Regarding secondary outcomes, only the 50- to 59-year-old group showed a significant survival difference between the two sexes (OR, 2.62 [95% CI, 1.21–5.69], *p* = 0.015). The trend of change in the adjusted OR for the survival discharge as the patients aged was similar to the trend for neurological outcomes. In contrast to the other age groups, males aged < 40 years were less likely to survive than females, although the difference was not significant (OR, 0.79 [95% CI, 0.31–2.00], *p* = 0.620).

### Neurological outcomes for each sex by age group

**Fig. 2:**
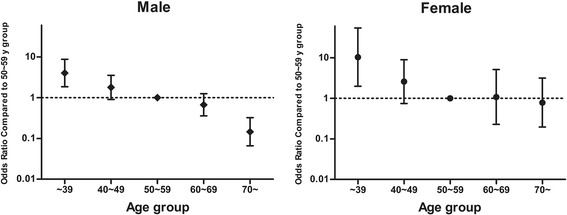
Adjusted ORs and 95% CIs for good neurological outcomes, stratified by sex and age group. The multivariate analysis controlled for the following risk factors: bystander-witnessed cardiac arrest, initial detected cardiac arrest rhythm, cardiac arrest cause, and anoxic time

In males, the adjusted OR for a good neurological outcome decreased from 4.03 (95% CI, 1.86–8.73) for those aged < 40 years to 1.79 (95% CI, 0.91–3.53) for those aged 40–49 years, 0.67 (95% CI, 0.36–1.25) for those aged 60–69 years, and 0.15 (95% CI, 0.07–0.32) for patients aged > 69 years when the 50- to 59-year-old age group was used as a reference (Fig. 2). In females, the adjusted ORs for a good neurological outcome were 10.34 (95% CI, 1.99–53.85), 2.59 (95% CI, 0.75–8.98), 1.08 (95% CI, 0.23–5.11), and 0.78 (95% CI, 0.20–3.14) for patients aged < 40 years, 40–49 years, 60–69 years, and ≥ 70 years, respectively.

## Discussion

We evaluated sex and age differences in neurological outcomes among adult OHCA patients treated with TTM. Age was associated with poor neurological outcomes, whereas sex was not associated with the neurological outcome. According to our results, males experienced a constant decrease in good neurological outcomes as they aged. In contrast, the outcomes of females worsened from ages 18 to 59 years of age and then remained steady.

Overall, males were significantly more likely to survive OHCA and have a good neurological outcome at discharge than females. A higher proportion of males also were witnessed during cardiac arrest, presented with ventricular fibrillation/ventricular tachycardia, and had presumed cardiac cause arrest. However, after adjustment for confounding variables through multivariate logistic regression, sex did not influence the neurological outcomes in these patients. This finding was consistent with previous studies of OHCA that included the entire EMS population [8, 9]. To evaluate the interactions between sex and age, we adjusted for covariates in the interaction model, and we found that sex did not influence the neurological outcome.

We also categorized patients by age and evaluated the effect of sex in different age subgroups. Being a female of reproductive age was not associated with neurological outcomes, and there was no survival difference between reproductive age females and their male counterparts. In comparison, survival to discharge at older ages was more common among the male group than among the female group. When we divided the patients into five age groups based on the age distribution of the enrolled cohort, the outcomes between sexes did not differ within the same age range, with the exception of survival in the 50- to 59-year-old group.

Many prior studies have suggested that endogenous estrogen is protective against the occurrence of OHCA [21–26] and might have cardioprotective effects for cardiac arrest [27, 28]. Other reports suggested a protective effect of endogenous estrogen against not only survival after cardiac arrest but also recovery from neurological outcomes [29, 30]. However, the impact of sex on neurological outcomes has not been consistent in different clinical studies. The ages of the female patients included in the studies was considered one important reason for this inconsistency. In our study, we evaluated the sex effect in specific age groups. In South Korea, the withdrawal of life-sustaining treatment for patients with a poor prognosis is not currently permitted, and survival to hospital discharge often does not concur with a good neurological outcome. This study may provide a more accurate estimate of the effect of estrogen on cardioprotection and/or neuroprotection in TTM-treated patients.

Although females aged < 40 years were more likely to survive than males of the same age (which was in contrast to the findings for other age groups), this difference was not statistically significant. In addition, this trend of survival in younger females was not consistent with the findings for the 40- to 49-year-old group. Therefore, our results do not provide evidence that endogenous estrogen has a cardioprotective effect in TTM-treated patients.

In contrast to previous studies regarding OHCA in which researchers enrolled the entire EMS population attended for cardiac arrest, in the reproductive age range, sex did not influence the neurological outcome. One explanation might be that in our study we examined TTM-treated patients. Considering that the leading neuroprotective mechanism of estrogen is anti-inflammatory, TTM and estrogen may appear to share the same neuroprotective mechanisms. In contrast to the experimental study, a neuroprotective effect of endogenous estrogen might be attenuated or underestimated in the human TTM setting using clinical measures for neurological function [31]. Our study is comparable to four studies that included TTM-treated OHCA patients who were similar to our study subjects [13–16]. In all studies, female sex was not associated with neurological outcomes when other confounders were adjusted, but the authors did not analyze the impact of reproductive age on the outcome [13–16]. To the best of our knowledge, the present study is the first clinical report to describe the sex-specific impact of reproductive age on neurological outcomes after TTM. In contrast to our cohort, which comprised relatively young patients, the small number of patients in these age groups who receive TTM may explain why this type of analysis is uncommon in the literature.

Another important finding of our study is the relationship between age and neurological outcomes after TTM. First, we showed that age was associated with a poor neurological outcome through multivariate logistic regression analysis. When we divided our cohort into five age subgroups, patients aged < 40 years had a significantly better outcome than patients of both sexes in the 50- to 59-year-old group. Males exhibited a constant decrease in the good neurological outcome rate as they aged. In contrast, the neurological outcomes in females did not exhibit a linear relationship with age. Instead, the neurological outcomes of females worsened from ages 18 to 59 years and then remained steadily constant. These findings are similar to those of a study by Arrich et al. in which a quartile of the included patients underwent TTM [32]. The reason why females but not males present a constant neurological outcome after the age of 50 years is unclear. Post hoc analysis of previous studies using similar subjects is needed to confirm our results.

Our finding has significant implications for future OHCA treatment strategies. In females with TTM, neurological outcomes may not be significantly influenced by advanced age. The effect of endogenous estrogen on these patients requires further analysis and evidence.

The results of this study should be cautiously interpreted in the context of several limitations. A major limitation of this retrospective study was the possibility of selection bias. Because our findings were not based on the enrollment of consecutive patients, any systematic bias in the indication for TTM between males and females could influence the differences between the two sexes, especially in patients aged ≥ 70 years. Second, the neurological outcomes in our study were evaluated upon hospital discharge. Neurological recovery after OHCA in elderly patients may be slower than recovery in younger populations. Therefore, we could not determine the impact of interactions between sex and age on the long-term neurological outcomes. Third, the number of patients in each age group might have been too small to draw conclusions about significant outcome differences between the sexes and age groups. In this retrospective study, the patients were stratified by sex and divided into five age groups. The wide CIs reflect the relatively small sample size. Finally, although most centers included the same TTM parameters, variable protocols or differences in care between hospitals could affect patients’ survival and neurological outcomes. Thus, our results require further confirmation in larger prospective studies.

## Conclusions

Overall, males with TTM were significantly more likely than females to survive OHCA and have a good neurological outcome at discharge. However, after adjustment for confounding variables, sex did not influence the neurological outcomes of these patients. When adjusted sex effects were examined by age subgroup, the neurological outcomes did not differ between sexes within the same age range. Age was associated with poor neurological outcomes. In our analysis of age subgroups, males exhibited a constant decrease in the good neurological outcome rate as they aged. In contrast, neurological outcomes in females worsened from ages 18 to 59 years and then remained constant.

## Abbreviations

CAG: Coronary angiography
CPC: Glasgow-Pittsburgh Cerebral Performance Categories
CPR: Cardiopulmonary resuscitation
EMS: Emergency medical service
KORHN: Korean Hypothermia Network
OHCA: Out-of-hospital cardiac arrest
ROSC: Return of spontaneous circulation
TTM: Targeted temperature management
